# Estimate of prevalent ischemic stroke from triglyceride glucose-body mass index in the general population

**DOI:** 10.1186/s12872-020-01768-8

**Published:** 2020-11-12

**Authors:** Zhi Du, Liying Xing, Min Lin, Yingxian Sun

**Affiliations:** 1 Department of Cardiovascular Medicine, The First Hospital of China Medical University, Shenyang, 110001 Liaoning China; 2 Disease Control and Prevention of Liaoning Province, Shenyang, Liaoning People’s Republic of China; 3 Department of Cardiovascular Medicine, Benxi Central Hospital, Benxi, Liaoning China

**Keywords:** Triglyceride glucose-body mass index, Ischemic stroke, Insulin resistance, Epidemiology

## Abstract

**Background:**

To investigate the relationship between triglyceride glucose-body mass index (TyG-BMI) and ischemic stroke.

**Methods:**

Leveraging two Chinese general population surveys, the Northeast China Rural Cardiovascular Health Study (NCRCHS, *N* = 11,097) and the National Stroke Screening and Intervention Program in Liaoning (NSSIPL, *N* = 10,862), we evaluated the relationship between TyG-BMI and ischemic stroke by a restricted cubic spline and multivariate logistic regression after adjusting age, sex, level of education, exercise regularly, current smoking, current drinking, atrial fibrillation, hypertension, coronary artery disease, low-density lipoprotein cholesterol, and high-density lipoprotein cholesterol. The category-free analysis was used to determine whether TyG-BMI enhanced the capacity of estimating ischemic stroke.

**Results:**

A total of 596 and 347 subjects, respectively, from NSSIPL and NCRCHS were survivors of ischemic stroke. In NSSIPL, the relationship between TyG-BMI and ischemic stroke was linear and did not have a threshold or saturation effect according to the results of the restricted cubic spline. The regression analysis indicated that the risk of ischemic stroke increased 20% for per SD increase of TyG-BMI after multivariate adjustment [odds ratio (OR): 1.20, 95% confidence interval (CI): 1.10–1.32]. Compared with those in the lowest tertile, the risk of ischemic stroke in subjects with intermediate and high TyG-BMI was significantly higher [OR (95% CI): 1.39 (1.10–1.74); OR (95% CI) 1.72 (1.37–2.17), respectively]. Category-free analysis indicated that TyG-BMI had a remarkable improvement in the ability to estimate prevalent ischemic stroke [NRI (95% CI): 0.188 (0.105–0.270)]. These abovementioned relationships were confirmed in NCRCHS.

**Conclusions:**

The present study found the robust correlation between TyG-BMI and ischemic stroke, independently of a host of conventional risk factors. Meanwhile, our findings also suggested the potential usefulness of TyG-BMI to improve the risk stratification of ischemic stroke.

## Background

As the leading cause of death and disability in most countries, stroke presents an increasing burden on the global healthcare system [1]. According to the Global Burden of Disease 2013 Study, there were approximately 25.7 million stroke survivors alive, 6.5 million stroke-related deaths and 113 million stroke-related disability-adjusted life-years worldwide [2]. Among all patients with incident stroke who survived or died, ischemic stroke is the most common pathological type [2, 3]. Although the age-standardized mortality rate of stroke has declined around the world, the incidence and prevalence of stroke are still explosively increasing in China [2–4]. It is generally known that smoking, hypertension, and diabetes are major risk factors for stroke [1, 4]. Worse outcomes are ordinarily found in patients without typical risk factors for stroke [5]. Therefore, there is an urgent need to develop some cost-effective and reproducible markers to improve stroke risk stratification, especially in ischemic stroke.

Insulin resistance (IR) is a frequent pathological condition in which cells have an impairment in the ability to respond to the hormone insulin. Accumulating evidence has revealed a significantly positive association between IR and ischemic stroke. Experimental studies have partially identified these mechanisms: atherosclerosis is a notoriously pathophysiological process of ischemic stroke, IR can accelerate the progression of atherosclerosis by decreasing eNOS activation (triggering endothelium dysfunction), increasing VCAM-1 expression (triggering leukocyte adhesion) in endothelial cells and migration and proliferation of vascular smooth muscle cells [6, 7]. Meanwhile, IR can potentially induce prolonged endoplasmic reticulum stress in macrophages and contribute to macrophage apoptosis, which subsequently results in plaque necrosis in advanced atherosclerosis [6, 8]. Epidemiological evidence also supports the association between IR and ischemic stroke [9–12]. Clinical trials have also suggested the effectiveness of insulin and insulin sensitizers in the treatment of ischemic stroke [13, 14]. Hence, early identification and early intervention of IR are of great clinical significance in reducing the risk of ischemic stroke. The current gold standard for diagnosing IR is the euglycemic-hyperinsulinemic clamp, but its application is confined by the complicated procedure, high cost, and ethical concerns [15].

Recently, Yu-Lin Ko has put forward a novel index named the Triglyceride Glucose-Body Mass Index (TyG-BMI), calculated as Ln [TG (mg/dl) * FBG (mg/dl)/2] * BMI (kg/m^2^), as a potential and straightforward marker for IR [16]. Previous studies have evaluated its usefulness to multiple IR-related clinical diseases [17–21]. Nevertheless, it remains unclear whether TyG-BMI can identify ischemic stroke, independently of other conventional risk factors at present. Accordingly, the purpose of the present study was to assess whether a higher level of TyG-BMI was associated with ischemic stroke in two surveys of two population-based samples in China.

## Methods

### Study participants

Northeast China Rural Cardiovascular Health Study (NCRCHS) was a population-based cross-sectional investigation, and the design, survey methods, and laboratory techniques have been described in previous studies [22, 23]. Using the method of multi-stage, stratified and cluster random sampling, 14,016 permanent residents in Dawa, Zhangwu and Liaoyang counties in Liaoning province were invited to participate in the study, and a total of 11,597 subjects aged ≥ 35 years completed the study from January 2013 to August 2013. In the present study, 482 participants were furtherly excluded for the reasons: lacking or unreadable electrocardiograms (ECGs, n = 238), and missing data (n = 244). Eventually, 11,097 (95.6%) subjects were analyzed. The study was approved by the Ethics Committee of China Medical University (Shenyang, China). Written informed consent was obtained from all participants.

The National Stroke Screening and Intervention Program in Liaoning (NSSIPL) was a cross-sectional study conducted in rural areas of Liaoning province from September 2017 to May 2018. The design and enrolment process of the study has been previously described [24, 25]. Briefly, the method of multi-stage, stratified, and cluster random sampling was adopted to randomly select four counties, including Liaoyang, Chaoyang, Donggang, and Lingyuan, from the central, eastern and western regions of Liaoning province, and 19 villages were involved. With the exception of those who were pregnant or had mental disorders, all permanent residents aged at least 40 in each village (n = 12,808) were eligible to participate, and a total of 10,926 (85.3%) completed the survey. Sixty-four participants were furtherly excluded for the following reasons: the absence of blood samples (n = 26) and abnormal or missing information (n = 38). Finally, data of 10,862 (99.4%) subjects were analyzed. The study was approved by the Central Ethics Committee at the China National Center for Cardiovascular Disease and received the written informed consent of all participants.

### Data collection and measurement

Data collection and anthropometric measurements were similar in NCRCHS and NSSIPL. Demographic and clinical information, including age, sex, socioeconomic status, lifestyle (smoking, drinking, and physical activity) were collected by a team of cardiologists, neuroscientists, and experts trained in chronic disease prevention and control. Self-administered questionnaires were fulfilled by face-to-face interviews during a clinical visit. Before the start of data collection, all team members were selected through a rigorous process, and pilot interviews with volunteers were completed.

If subjects answered “yes” to the question that “Have you been diagnosed with [specific disease] by a registered physician?”, then they were identified as having complications including hypertension, dyslipidemia, diabetes, and atrial fibrillation or a history of a specific disease such as diabetes, coronary heart disease, (CHD) or cerebrovascular disease. Subsequently, the subjects with self-reported complications and specific diseases were asked if they had taken prescription drugs to control the disease in the past 2 weeks. Those who answered “yes” were asked to report the name, dose, and frequency of each drug if known. Those who did not remember the exact dose were asked the number of pills taken.

Physical measurements were obtained, including height and weight. The weight and height were measured to the nearest 0.1 kg and 0.1 cm, respectively, with participants wearing light clothing and no shoes. The body mass index (BMI) was defined as weight in kilograms divided by the square of the height in meter. After sitting for at least 5 min, each participant was measured blood pressure three times at 2 min intervals with a standardized, automated electronic sphygmomanometer (HEM-907; Omron, Tokyo, Japan in NCRCHS; J30; Omron, Kyoto, Japan in NSSIPL). A central steering committee with a subcommittee for quality control was established to ensure that data were obtained according to standardized protocols.

Blood samples were taken from participants who fasted for at least 8 h. The samples were taken from an antecubital vein using EDTA (BD Vacutainer tubes containing ethylenediaminetetraacetic acid; Becton, Dickinson and Co., Franklin Lakes, NJ, USA), and serum was subsequently isolated from the whole blood. Thereafter, the serum samples were cryopreserved at − 20 °C. Subsequently, the biochemical parameters including fasting blood glucose (FBG), glycosylated hemoglobin (HbA1c), triglyceride (TG), total cholesterol (TC), low-density lipoprotein cholesterol (LDL-C), and high-density lipoprotein cholesterol (HDL-C) were measured by an Olympus AU640 Auto-Analyzer (Olympus Corp., Kobe, Japan) in NCRCHS and an Abbott Diagnostics C800i auto-analyzer (Abbott Laboratories, Abbott Park, IL, USA) in NSSIPL, respectively. Because Laboratory tests were conducted in three laboratories in NSSIPL, we randomly selected 10% samples from each laboratory for centralized retesting by China’s Ministry of Health’s National Center for Clinical Laboratories to ensure the accuracy of the test.

### Definition

TyG-BMI was calculated as follows: TyG-BMI = Ln [TG (mg/dl) * FBG (mg/dl)/2] * BMI (kg/m^2^) [16]. Ischemic stroke was diagnosed by a neurologist on the recommendation of the World Health Organization and confirmed by computed tomography (CT) and/or magnetic resonance imaging (MRI) [26]. Hypertension was determined as mean systolic blood pressure (SBP) ≥ 140 mmHg or mean diastolic blood pressure (DBP) ≥ 90 mmHg and/or self-reported use of antihypertensive drugs in the past 2 weeks [27]. Dyslipidemia was diagnosed if the participants met one of the following criteria: (1) serum TC level ≥ 6.22 mmol/L; (2) serum LDL-C level ≥ 4.14 mmol/L; (3) serum TG level ≥ 2.26 mmol/L; (4) serum HDL-C level < 1.04 mmol/L; and (5) self-reported use of lipid-regulating drugs [28]. Diabetes mellitus was defined as FBG ≥ 7.0 mmol/L or HbA1c ≥ 6.5%, and/or self-reported diabetes [29]. AF was diagnosed by a physician based on an electrocardiogram and/or previous diagnosis. Regular exercise was defined as moderate-intensity exercise (equivalent to walking) ≥ 30 min and ≥ 3 times per week.

### Statistical analysis

Level of TyG-BMI was divided into three categories (low, intermediate, and high) by tertiles in NSSIPL. Then, according to the categories of TyG-BMI, continuous variables were presented as means and standard deviations and categorical variables were reported as frequencies and percentages in each subgroup. One way analysis of variance (ANOVA) and chi-squared tests were used, as appropriate, to compare differences among the 3 groups. The dose–response relationship between TyG-BMI and the risk of ischemic stroke was evaluated by a restricted cubic spline. Meanwhile, multivariate logistic regression analysis was carried out to evaluate the association between TyG-BMI and ischemic stroke. As a categorical variable, or a continuous variable using the standard deviation transformed, level of TyG-BMI was incorporated separately into regression model analysis, adjusting for conventional risk factors, including age, sex, level of education, exercise regularity, current smoking, current drinking, AF, hypertension, coronary artery disease, LDL-C, and HDL-C. Odds ratios (ORs) and 95% confidence intervals (CIs) were presented for the logistic regression analyses. TyG-BMI was derived from FBG, TG, and BMI. Therefore, to examine whether TyG-BMI was indeed an independent risk factor and whether the association between TyG-BMI and ischemic stroke depended entirely on diabetes, obesity or overweight, dyslipidemia. We additionally adjusted for diabetes, obesity or overweight, and dyslipidemia separately or simultaneously. To evaluate the extent to which TyG-BMI improved prediction performance over conventional risk factors, net reclassification improvement (NRI) was calculated and compared in NSSIPL (conventional risk factors only vs. conventional risk factors + TyG-BMI). Taking the NSSIPL as an example, the NCRCHS data was analyzed using the same methods to verify the conclusion of the NSSIPL. Statistical analyses were performed using SPSS software version 22.0 (SPSS Inc., Chicago, IL, USA), EmpowerStats (https://www.empowerstats.com, X&Y Solutions, Inc., Boston, MA) and statistical software packages R (https://www.R-project.org, The R Foundation); *p* values < 0.05 were considered statistically significant.

## Results

Our study included 10,862 NSSIPL subjects (mean age: 59.95 ± 10.06, males: 40.2%) and 11,097 NCRCHS participants (mean age: 53.83 ± 10.56, males: 45.6%). Clinical characteristics by level of TyG-BMI in two surveys were shown in Tables 1 and 2, respectively. The average TyG-BMI level of individuals in the two cross-sections was analogous (means ± standard deviations: 218.24 ± 40.83 in NSSIPL; 216.74 ± 40.17 in NCRCHS). 596 of the 10,896 NSSIPL subjects (5.4%) and 347 of the 11,097 NCRCHS participants (3.1%) were survivors of ischemic stroke. Compared with subjects with lower levels of TyG-BMI, individuals with higher TyG-BMI had significantly higher SBP, DBP, FBG, TG, TC, LDL-C, and lower HDL-C in both NSSIPL and NCRCHS (all *ps* < 0.05). In addition, subjects with higher levels of TyG-BMI had a significantly higher prevalence of overweight or obesity, diabetes, dyslipidemia, CHD, and ischemic stroke (all *ps* < 0.05).

**Table 1:** Baseline characteristics of the NSSIPL participants according to TyG-BMI levels

Characteristics	Level of TyG-BMI			
	Low (*N* = 3617)	Intermediated (*N* = 3628)	High (*N* = 3617)	*p* value for liner trend
Age (years)	61.64 ± 10.82	59.63 ± 9.81	58.57 ± 9.26	< 0.001
Male [n (%)]	1768 (48.9)	1381 (38.1)	1215 (33.6)	< 0.01
Education [n (%)]				< 0.001
Primary school or below	2209 (61.1)	2135 (58.8)	2080 (57.5)	
Middle school	1140 (31.5)	1165 (32.1)	1169 (32.3)	
High school or higher	268 (7.4)	328 (9.0)	368 (10.2)	
Current smoking [n (%)]	1305 (36.1)	881 (24.3)	687 (19.0)	< 0.001
Current drinking [n (%)]	1188 (32.8)	969 (26.7)	900 (24.9)	< 0.001
Regular exercise [n (%)]	2979 (82.4)	3089 (85.1)	2997 (82.9)	0.569
SBP (mmHg)	139.50 ± 23.24	145.70 ± 22.47	151.88 ± 23.50	< 0.001
DBP (mmHg)	83.31 ± 11.57	86.45 ± 11.28	90.28 ± 11.88	< 0.001
FBG (mmol/L)	5.64 ± 1.16	6.06 ± 1.59	6.78 ± 2.42	< 0.001
TG (mmol/L)	0.98 ± 0.45	1.50 ± 0.89	2.48 ± 2.15	< 0.001
TC (mmol/L)	4.79 ± 0.98	5.14 ± 1.09	5.41 ± 1.20	< 0.001
LDL-C (mmol/L)	2.11 ± 0.80	2.37 ± 1.00	2.58 ± 1.18	< 0.001
HDL-C (mmol/L)	2.07 ± 0.61	1.94 ± 0.74	1.86 ± 0.88	< 0.001
Obesity/overweight [n (%)]	26 (0.7)	1542 (42.5)	3397 (93.9)	< 0.001
Hypertension [n (%)]	1757 (48.6)	2185 (60.2)	2636 (72.9)	< 0.001
Diabetes [n (%)]	217 (6.0)	508 (14.0)	1033 (28.6)	< 0.001
Dyslipidemia [n (%)]	422 (11.7)	998 (27.5)	1946 (53.8)	< 0.001
Atrial fibrillation [n (%)]	49 (1.4)	33 (0.9)	35 (1.0)	0.111
CHD [n (%)]	123 (3.4)	135 (3.7)	197 (5.4)	< 0.001
Ischemic stroke [n (%)]	159 (4.4)	194 (5.3)	243 (6.7)	< 0.001

*NSSIPL* National Stroke Screening and Intervention Program in Liaoning, *TyG-BMI* triglyceride glucose-body mass index, *SBP* systolic blood pressure, *DBP* diastolic blood pressure, *FBG* fasting blood glucose, *TG* triglycerides, *TC* total cholesterol, *LDL-C* low-density lipoprotein cholesterol, *HDL-C* high-density lipoprotein cholesterol, *CHD* coronary heart disease

**Table 2:** Baseline characteristics of the NCRCHS participants according to TyG-BMI levels

Characteristics	Level of TyG-BMI			
	Low (*N* = 3695)	Intermediated (*N* = 3708)	High (*N* = 3695)	*p* value for liner trend
Age (years)	53.79 ± 11.18	53.81 ± 10.49	53.89 ± 9.99	0.677
Male [n (%)]	1738 (47.0)	1669 (45.0)	1654 (44.8)	0.050
Education [n (%)]				0.522
Primary school or below	1802 (48.8)	1869 (50.4)	1876 (50.8)	
Middle school	1578 (42.7)	1476 (39.8)	1466 (39.7)	
High school or higher	315 (8.5)	362 (9.8)	353 (9.5)	
Current smoking [n (%)]	1532 (41.5)	1229 (33.2)	1134 (30.7)	< 0.001
Current drinking [n (%)]	833 (22.5)	831 (22.4)	806 (21.8)	0.450
Regular exercise [n (%)]	2676 (72.4)	2668 (72.0)	2471 (66.9)	< 0.001
SBP (mmHg)	134.54 ± 21.50	141.71 ± 23.01	148.81 ± 23.57	< 0.001
DBP (mmHg)	78.09 ± 10.80	81.94 ± 11.31	85.98 ± 11.82	< 0.001
FBG (mmol/L)	5.49 ± 0.93	5.77 ± 1.32	6.45 ± 2.20	< 0.001
TG (mmol/L)	0.97 ± 0.44	1.40 ± 0.80	2.55 ± 2.15	< 0.001
TC (mmol/L)	4.93 ± 0.97	5.22 ± 1.03	5.55 ± 1.16	< 0.001
LDL-C (mmol/L)	2.64 ± 0.71	2.95 ± 0.78	3.18 ± 0.88	< 0.001
HDL-C (mmol/L)	1.55 ± 0.41	1.42 ± 0.37	1.26 ± 0.29	< 0.001
Obesity/overweight [n (%)]	26 (0.7)	1470 (39.7)	3471 (93.9)	< 0.001
Hypertension [n (%)]	1325 (35.9)	1884 (50.8)	2449 (66.3)	< 0.001
Diabetes [n (%)]	128 (3.5)	272 (7.3)	752 (20.4)	< 0.001
Dyslipidemia [n (%)]	674 (18.2)	1315 (35.5)	2406 (65.1)	< 0.001
Atrial fibrillation [n (%)]	42 (1.1)	43 (1.2)	53 (1.4)	0.248
CHD [n (%)]	144 (3.9)	171 (4.6)	258 (7.0)	< 0.001
Ischemic stroke [n (%)]	68 (1.8)	99 (2.7)	180 (4.9)	< 0.001

Abbreviations as in Table 1; *NCRCHS* Northeast China Rural Cardiovascular Health Study

**Fig. 1:**
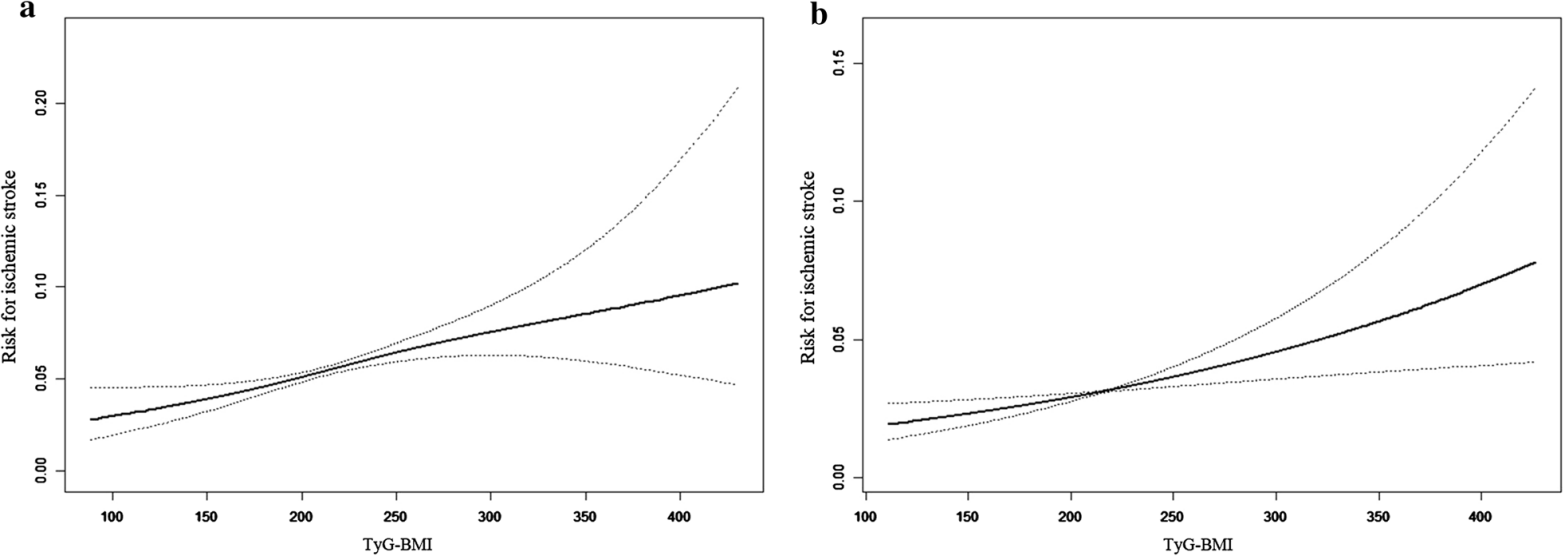
The associated between TyG-BMI and ischemic stroke in NSSIPL (**a**) and NCRCHS (**b**) by restricted cubic spline

The linear associations were demonstrated between the level of TyG-BMI and ischemic stroke in two surveys (Fig. 1). The risk of ischemic stroke obviously increased with the level of TyG-BMI. Further, the relationships were assessed separately by the multivariate logistic regression models in both surveys, and results were shown in Table 3. In NSSIPL, the regression analysis using the standard deviation transformed TyG-BMI as a continuous variable indicated that the risk of ischemic stroke increased 20% per SD increase after multivariate adjustment [OR: 1.20, 95% CI: 1.10–1.32]. Then, as a categorical variable (low vs. intermediate vs. high) brought into the regression equation, TyG-BMI implied a similar correlation with ischemic stroke. Participants with low levels of TyG-BMI were used as a reference, and the risk of ischemic stroke in subjects with intermediate and high TyG-BMI was significantly higher [OR (95% CI): 1.39 (1.10–1.74) in the intermediate TyG-BMI group; OR (95% CI) 1.72 (1.37–2.17) in the high TyG-BMI group]. The linkage between TyG-BMI and ischemic stroke did not change after additionally adjusting for diabetes (OR: 1.34, *p* = 0.012; OR: 1.58, *p* < 0.001) or obesity or overweight (OR: 1.44, *p* = 0.004; OR: 1.89, *p* < 0.001). Even if we adjusted diabetes, obesity or overweight, and dyslipidemia simultaneously, the correlation between TyG-BMI and stroke still existed in the high-level group (OR: 1.30, *p* = 0.044; OR: 1.48, *p* = 0.033). These conclusions were confirmed among participants from NCRCHS.

**Table 3:** The association between TyG-BMI and ischemic stroke by multivariate logistic regression analysis

TyG-BMI	Sensitive analysis							
	Mode 1 OR (95% CI)	*P* value	Mode 2 OR (95% CI)	*P* value	Mode 3 OR (95% CI)	*P* value	Mode 4 OR (95% CI)	P value
NSSIPL (N = 10,862)								
Continuous								
Per SD increase	1.20 (1.10–1.32)	< 0.001	1.16 (1.06–1.27)	0.002	1.20 (1.07–1.36)	0.003	1.07 (0.93–1.23)	0.324
Categorical								
Low	1	–	1	–	1	–	1	–
Intermediated	1.39 (1.10–1.74)	0.005	1.34 (1.07–1.69)	0.012	1.44 (1.12–1.85)	0.004	1.30 (1.01–1.68)	0.044
High	1.72 (1.37–2.17)	< 0.001	1.58 (1.25–2.01)	< 0.001	1.89 (1.35–2.64)	< 0.001	1.48 (1.03–2.13)	0.033
NCRCHS (*N* = 11,097)								
Continuous								
Per SD increase	1.21 (1.07–1.36)	0.002	1.17 (1.03–1.33)	0.015	1.25 (1.06–1.48)	0.008	1.13 (0.96–1.35)	0.170
Categorical								
Low	1	–	1	–	1	–	1	–
Intermediated	1.22 (0.88–1.71)	0.240	1.21 (0.87–1.69)	0.262	1.37 (0.97–1.95)	0.076	1.29 (0.91–1.83)	0.152
High	1.84 (1.32–2.56)	< 0.001	1.75 (1.25–2.45)	0.001	2.58 (1.62–4.13)	< 0.001	2.12 (1.32–3.41)	0.002

Model 1: Adjusting for age, sex, education, regular exercise, current smoking, current drinking, atrial fibrillation, hypertension, coronary heart disease, LDL-C, HDL-C

Model 2: Adjusting for all risk the factors in model 1 and diabetes

Model 3: Adjusting for all risk the factors in model 1 and overweight or obesity

Model 4: Adjusting for age, sex, education, regular exercise, current smoking, current drinking, atrial fibrillation, hypertension, coronary heart disease, overweight or obesity and dyslipidemia

*OR* odds ratio, *CI* confidence interval, *SD* standard deviation, *NSSIPL* National Stroke Screening and Intervention Program in Liaoning; *NCRCHS* Northeast China Rural Cardiovascular Health Study

The category-free analysis was performed to evaluate the effect that TyG-BMI improved the risk stratification of ischemic stroke in both NSSIPL and NCRCHS (Table 4). Compared with conventional risk factors only, analysis results showed that the ability to predict ischemic stroke was remarkably improved after adding TyG-BMI [NRI (95% CI): 0.188 (0.105–0.270) in NSSIPL; 0.197 (0.091–0.304) in NCRCHS].

**Table 4:** The value that TyG-BMI improved risk stratification of ischemic stroke according to NRI

Variable	NSSIPL (*N* = 10,862)	*P* value	NCRCHS (*N* = 11,098)	*P* value
Conventional risk factors	reference		Reference	
Conventional risk factors + TyG-BMI	0.188 (0.105–0.270)	< 0.001	0197 (0.091–0.304)	< 0.001

Conventional risk factors include age, sex, education, regular exercise, current smoking, current drinking, atrial fibrillation, hypertension, coronary heart disease, LDL-C, HDL-C

*TyG-BMI* triglyceride glucose-body mass index, *NRI* net reclassification improvement, *NSSIPL* National Stroke Screening and Intervention Program in Liaoning, *NCRCHS* Northeast China Rural Cardiovascular Health Study

## Discussion

Our study demonstrated that a significantly positive association existed between TyG-BMI and ischemic stroke in two surveys based on the general population. The relationship was linear and independent of a host of conventional risk factors. We also noticed that the effect of TyG-BMI on ischemic stroke was exerted beyond diabetes, obesity, and dyslipidemia, although TyG-BMI was calculated from FBG, TG, and BMI. Meanwhile, our results suggested that TyG-BMI significantly improved the risk stratification of ischemic stroke. In brief, the results of our research provided clinical guidance that TyG-BMI was a potential marker to estimate prevalent ischemic stroke.

A previous study has proved that high levels of free fatty acids in plasma can rapidly give rise to IR in humans [30]. Meanwhile, under high-glucose conditions, glucose molecules conjugate to proteins similar to the insulin receptor on the surface of the cytoplasmic membrane, then convert to advanced glycation endproducts promptly [31]. Serum insulin cannot fit perfectly with the advanced glycation endproducts deposited on the insulin receptor and then fail to mediate insulin's stimulation of glucose transport, triggering IR [32, 33]. Recently, some studies have suggested that the product of triglycerides and glucose in plasma (TyG) is effective in detecting IR [34, 35]. Besides, BMI is a simple anthropometric parameter, which is often used as an index of obesity and IR. In obese individuals, adipose tissue lipolysis increases and releases considerable free fatty acids, which is perhaps the most paramount element in modulating insulin sensitivity [36, 37]. Therefore, there is a reasonable hypothesis that TyG-BMI, based on the anthropometric BMI and TyG parameters, also serve as markers for IR.

In fact, the correlation between TyG-BMI and homeostatic model assessment (HOMA)-IB has been proven [16]. Subsequently, TyG-BMI was recommended as a marker to evaluate IR and IR-related diseases in many studies. A cross-sectional study conducted in rural Beijing, China confirmed that TyG-BMI was a better indicator in detecting IR [38]. The 2015 Health, Well-Being, and Aging Study also suggested that TyG-BMI could be used to assess prediabetes, although it was not the optimal index [17]. Results from a large cross-sectional survey involving 11,149 participants, the Korean National Health and Nutrition Examination Survey, indicated that TyG-BMI was an alternative marker for evaluating IR after comparing with other parameters of IR [39]. Besides, these associations between TyG-BMI with prehypertension and hypertension have been confirmed.[20, 21] However, the relationship between TyG-BMI and ischemic stroke remains unclear. To obtain the accurate association, other conventional risk factors including age, sex, level of education, exercise regularity, current smoking, current drinking, AF, hypertension, coronary artery disease, LDL-C, and HDL-C were thoroughly adjusted in our analysis, and results indicated that high levels of TyG-BMI was a risk factor for ischemic stroke. The relationship was linear and did not have a threshold or saturation effect according to the results of the restricted cubic spline. TyG-BMI was calculated by FBG, TG, and BMI, but the observed relationship between TyG-BMI and ischemic stroke was unlikely to be confounded by associated clinical diseases because diabetes, obesity or overweight, and dyslipidemia were adjusted in the additional analysis at the same time. This also implied that TyG-BMI might be an alternative indicator of IR to predict ischemic stroke independently.

When adding new markers to existing prediction models, it is necessary to calculate NRI to determine whether the new assessment model is meaningful [40]. Fortunately, category-free analysis inferred that this significantly enhanced capacity of estimating ischemic stroke when adding TyG-BMI to conventional risk factors in the two investigations. The NRI value suggested that the model after addition of TyG-BMI led to a significant improvement in the risk stratification of ischemic stroke.

Our study provided the reference for the application of TyG-BMI as a clinically useful marker in identification of individual at high risk for cardiovascular disease. Tyg-BMI was calculated by FBG, TG and BMI. Obviously, these biochemical parameters can be obtained from a single sample at the same time, which is cheap and convenient compared to euglycemic-hyperinsulinemic clamp. Besides, in terms of generality, height, weight, FBG and TG are routinely performed biochemical tests in primary hospitals, which is advantage for clinical and epidemiological studies. Hence, we have reason to believe that Tyg-BMI has good application prospects to identify patients with high risk of cardiovascular disease.

The prevalence of ischemic stroke in our populations performed “significantly above” that of the 2014 national level in participants aged 40 and older (3.1% vs. 1.8% or 5.4% vs. 1.8%) [4]. However, this was reasonable due to our special samples, which were composed of rural population aged ≥ 40 years in northeast China. Previous research demonstrated that stroke distributed in a north-to-south gradient in China and the northern region suffered from the highest stroke burden. Simultaneously, the prevalence of stroke in rural residents was significantly higher than that in urban residents [3]. Although participants in both NSSIPL and NCRCHS hailed from northeast China, the individuals in NSSIPL were older and had higher prevalence of hypertension and diabetes. As for age, Michael et al. [41] asserted that increased risk for stroke rose more than doubles for each successive 10 years after the age of 55. Hypertension and diabetes had invariably been well-documented risk factors for ischemic stroke [4]. Additionally, the prevalence of stroke presented an escalating trend in China, and NCRCHS was conducted 5 years ahead of NSSIPL [3]. To sum up, it was accepted that the prevalence of ischemic stroke in NSSIPL was higher than that in NCRCHS.

The present study indicated the linear association of TyG-BMI with ischemic stroke. The strengths of our results were repetitive in two surveys. However, there were some limitations in our study. Firstly, the cross-sectional design of the study only allowed assessment of the associations between TyG-BMI and ischemic stroke rather than causal links. Secondly, although our studies contained a large number of individuals, they were from northeast China, over 40 years old, and mostly postmenopausal women. This might reduce the ability of TyG-BMI to estimate ischemic stroke and restrict applicability of our results to other populations. Thirdly, the comorbidities and medical history of participants were self-reported in our study, which might lead to false-positive results. Subsequently, some confounding factors proved in other studies, such as inflammation, were not collected in our study. Finally, recruiting only ischemic stroke survivors might affect the accuracy of our results.

## Conclusions

Our results suggested the independent association between TyG-BMI and ischemic stroke in the general population and the relationship was linear and did not have a threshold or saturation effect. Meanwhile, our study suggests the potential usefulness of TyG-BMI to improve the risk stratification of ischemic stroke.

## Data Availability

The datasets used and/or analysed during the current study are de-identified and available from the corresponding author on reasonable request.

## Abbreviations

BMI: Body mass index
CHD: Coronary heart disease
DAG: Diacylglycerol
DBP: Diastolic blood pressure
FBG: Fasting blood glucose
HbA1c: Glycosylated hemoglobin
HDL-C: High-density lipoprotein cholesterol
IR: Insulin resistance
LDL-C: Low-density lipoprotein cholesterol
NCRCHS: Northeast China Rural Cardiovascular Health Study
NSSIPL: The National Stroke Screening and Intervention Program in Liaoning
PKCε: Protein kinase C epsilon
SBP: Systolic blood pressure
TC: Total cholesterol
TG: Triglyceride
TyG-BMI: Triglyceride glucose-body mass index
